# Black carbon emissions in Jordan: national inventory, climate and health implications (2022–2050)

**DOI:** 10.1088/2515-7620/ae1660

**Published:** 2025-11-04

**Authors:** Alham Al-Shurafat, Fayez Abdulla, Ayman Sharafat, Anas Ghawanmeh

**Affiliations:** 1 Department of Civil, Construction, and Environmental Engineering, North Carolina State University (NCSU), Raleigh, NC, United States of America; 2 Department of Civil Engineering, Jordan University of Science and Technology (JUST), Irbid, Jordan; 3 Climate Action Association (CAN), Amman, Jordan

**Keywords:** LEAP-IBC, health implications, black carbon, Jordan, mitigation potential

## Abstract

Black carbon (BC), a short-lived climate pollutant with a global warming potential hundreds of times greater than CO_2_, remains largely excluded from international climate frameworks and is absent from Jordan’s national emission inventories. This study develops the first nationally validated BC inventory for Jordan, projects emissions to 2050, and evaluates associated health and climate impacts alongside mitigation potential using the Low Emissions Analysis Platform–Integrated Benefits Calculator (LEAP-IBC). Emission factors were locally validated through literature review and consultation with more than 40 national experts, replacing generic global defaults. The results estimate Jordan’s 2022 BC emissions at 6,977 tonnes (95% CI: 2,582–7,460 tonnes), with the transport sector contributing 59% ± 8% and the energy sector accounting for the remainder, both dominated by diesel combustion. Comparison with global datasets shows systematic underestimation of national emissions by factors of 2.8–4.5, consistent with findings for other countries. BC climate impacts are found to vary substantially with the choice of GWP metric: 2022 emissions are estimated at 2,480–23,004 GgCO_2_-eq, with GWP_(20)_ assumptions (1,200–3,200) producing higher estimates than GWP_(100)_ assumptions (345–900).. By 2050, BC climate impacts are projected at 6,034–55,970 GgCO_2_-eq, with GWP_(100)_ yielding lower values (6,034–15,742 GgCO_2_-eq) than GWP_(20)_ as its impact is diluted when averaged over 100 years. Model projections suggest that BC’s share of PM_2.5_ exposure will nearly double from 1.7% to 3.3% by 2050, with more than 90% of the associated health burden linked to cardiovascular diseases in an aging population. Coordinated mitigation across the transport and energy sectors could reduce BC emissions by up to 53% relative to baseline scenario, with priority strategies including Euro VI vehicle standards, public transport electrification, industrial efficiency improvements, and accelerated renewable energy deployment.

## 1. Introduction

Black carbon (BC), produced through the incomplete combustion of carbonaceous fuels including fossil fuels and biomass, is recognized as a major short-lived climate pollutant (SLCP) that exerts disproportionate effects on the climate system, air quality, agricultural productivity, and human health (Blanco-Donado *et al* 2022). BC has an atmospheric lifetime of only a few days to weeks (CCAC 2025) yet is estimated to account for nearly one-third of present global warming (World Economic Forum 2024). BC’s strong climate impact arises from its high absorptivity of solar radiation which indirectly warms surfaces, with global warming potential (GWP) hundreds of times greater than CO_2_ and an estimated radiative forcing of 0.28–0.41 W m^−12^, contributing roughly 0.1 °C to global warming since 1750 (World Economic Forum 2024). Its deposition on cryosphere surfaces reduces albedo by darkening snow and ice, thereby accelerating melting and amplifying Arctic warming, which occurs at a rate of about four times faster than the global average (Ohata *et al* 2021, Rantanen *et al* 2022). BC also modifies cloud formation and properties, although its net radiative effect on clouds remains highly uncertain (Stohl *et al* 2015). BC, a major component of fine particulate matter (PM_2.5_; aerodynamic diameter ≤ 2.5 μm) (Liu *et al* 2020), is associated with serious adverse health outcomes, including elevated blood pressure (Baumgartner *et al* 2014), increased transmission of infectious diseases (Hussey *et al* 2017), and increased mortality (Li *et al* 2016). Globally, household air pollution from solid fuels and kerosene combustion is responsible for an estimated 3.2 million premature deaths each year (WHO 2023). Beyond health, BC contributes to visibility degradation, disrupts regional rainfall patterns, and reduces agricultural productivity through leaf deposition and surface heating (Novakov and Rosen 2013). Thus, despite its relatively short atmospheric lifetime, reducing BC emissions delivers rapid and simultaneous benefits for climate, public health, and ecosystems (Wang *et al* 2023).

Rapid mitigation of BC and methane could reduce projected warming by up to 0.5 °C by 2050, preventing 2.4 million premature deaths each year and avoiding 52 million metric tons of crop losses by 2030 (Hussein *et al* 2019). Nevertheless, BC remains largely absent from international climate governance frameworks, including the six greenhouse gases regulated under the UNFCCC (Malley *et al* 2023). Only a limited number of countries explicitly address BC within their Nationally Determined Contributions (NDCs) or climate policies (World Economic Forum 2024). In Jordan, despite adherence to NDC commitments, BC mitigation is not included, reflecting the broader absence of national-scale BC inventories, projections, and impact assessments.

Early work by Hamasha *et al* (2010) and Hamasha and Arnott (2010) measured BC light absorption coefficients at selected sites in Jordan, while other studies such as Hussein *et al* (2017, 2018, 2019) examined urban aerosols and spatial BC/PM distribution in few cities. Satellite-derived observations from 2007–2018 showed a gradual increase in the amount of light blocked or absorbed by BC in the atmosphere over four Jordanian cities, which indicates slightly worsening BC pollution levels over time (Hamasha 2021). Most recently, Mallak *et al* (2024) identified fossil fuel combustion as the dominant source of BC in Al-Ramtha, Jordan, reporting concentrations of 2.06 μg m^−3^ that were strongly shaped by local geography and meteorological conditions. Collectively, these studies provide important groundwork; however, BC research in Jordan remains limited to site-specific assessments and lacks the spatial coverage, sectoral resolution, and temporal depth necessary to inform comprehensive national climate and health policies.

Global emission inventories such as EDGAR v8.1 (Crippa *et al* 2016), CEDS (Hoesly and Smith 2024), and ECLIPSE/GAINS (UNEP and WMO 2011, Klimont *et al* 2017) provide sectoral baseline estimates of BC emissions, but their application to Jordan exposes critical limitations that underscore the need for a nationally validated inventory. Our assessment indicates that EDGAR v8.1 and CEDS, while offering sector-resolved annual estimates since 1970, rely predominantly on IEA and FAO activity data without incorporating local validation. The GAINS/ECLIPSE modeling framework provides another global benchmark by generating gridded BC emission projections under various policy scenarios (e.g., current legislation baseline) from ∼1990 through 2050. Although valuable for policy analysis, its outputs are constrained by a five-year temporal resolution and dependence on default emission factors, which introduces considerable uncertainty—particularly for small-scale combustion, a major emission source in Jordan. Consequently, local authorities and researchers view these datasets as unreliable, since they provide only sector-level estimates, lack fuel-specific resolution, too low emission factors and report emission levels that appear substantially lower than observed air pollution in Jordan.

To address these gaps, this study develops the first national BC inventory for Jordan, providing policymakers and researchers with essential evidence to design targeted mitigation and health-protection strategies. The inventory introduces three major advancements: (1) activity data and emission factors validated by the Jordanian Ministry of Environment (MoEnv) in collaboration with more than 40 national experts from relevant institutions, replacing generic defaults; (2) alignment with national policies and reporting obligations to the Climate and Clean Air Coalition (CCAC); and (3) sectoral and fuel-level resolution tailored to Jordan’s context, as the initiative commissioned under the project ‘Jordan - Deliver policy analysis and recommendations on SLCP mitigation’ (CCAC 2023) in collaboration with the MoEnv. Official SLCP/BC inventory compiles national data for 2019–2022 and provides projections to 2050. It covers the energy sector—including residential, industrial, informal, agricultural, and waste-related fuel use—as well as transport, with explicit fuel-specific resolution. Incorporating fuel-level detail improves accuracy and reduces uncertainty, particularly for residential combustion and transport, where global inventories often exhibit ±45%–185% uncertainty due to assumptions in activity data and conservative emission factors (Klimont *et al* 2017).

This study also explores the application of the Low Emissions Analysis Platform–Integrated Benefits Calculator (LEAP-IBC) (SEI US 2025), replacing global default inputs with stakeholder-vetted input to develop a BC inventory that is validated locally and benchmarked against global datasets, thereby directly informing national climate policy. Previous national LEAP-IBC applications have focused primarily on South and South-East Asia—for example, Bangladesh (Kuylenstierna *et al* 2020); and Nepal (Nakarmi *et al* 2020) leaving a recognized gap for its application in other regions. Earlier studies typically relied on international activity datasets (e.g., IEA, FAO) and default emission factors, which introduced substantial uncertainty in fuel consumption estimates and open burning sources (Kuylenstierna *et al* 2017). In contrast, this study convened experts to compile bottom-up activity data and to reach consensus on emission factors reflecting national fuel quality, combustion conditions, and technology shares. This stakeholder-driven approach reduces input uncertainty while simultaneously strengthening in-country capacity for regular inventory updates. To our knowledge, no prior LEAP-IBC study has systematically compared national results against leading global BC datasets. This benchmarking both validates our national estimates and highlights areas where global models misrepresent local realities. Taken together, these innovations—including the first regional application, locally vetted inputs, fuel level, sectoral and temporal coverage with uncertainty analysis, and systematic comparison with global datasets—distinguish this work from earlier LEAP-IBC applications and provide an evidence base for Jordan’s forthcoming SLCPs national action plan and NDCs update.

## 2. Methodology and data

This study quantifies Jordan’s BC emissions, projects their trajectories, evaluates associated health and climate impacts, and assesses the mitigation potential of national climate policies. The methodology combines data collection, stakeholder engagement, and modeling using LEAP-IBC (SEI US 2025) at the sectoral and fuel level. LEAP-IBC was selected for its integrated capacity to model energy demand, SLCP/GHG emissions, and related health and climate impacts within a single transparent framework. Its graphical user interface allows Jordanian line ministries to update input data, reinforcing institutional ownership and enabling staff to continuously update the model while building in-house capacity (Kuylenstierna *et al* 2017). The policy utility of LEAP-IBC is demonstrated by national SLCP action plans in India, Russia, Ghana, Bangladesh, Nepal, Dominican Republic, China, and the United States (Paliwal, Sharma and Burkhart 2016, Evans *et al* 2017, Ghana Environmental Protection Agency 2018, Kuylenstierna *et al* 2020, Nakarmi *et al* 2020, Ministry of Environment of Dominican Republic 2021, Wang *et al* 2023, EPA US 2025a) with explicit endorsement by the CCAC Supporting National Action and Planning (SNAP) initiative. In Jordan, MoEnv has previously applied LEAP-IBC to develop methane and HFC inventories for the baseline years 2010 and 2016 (MoEnv 2017, 2020a). LEAP-IBC’s predictive reliability has also been validated across multiple national contexts: in Bangladesh, baseline PM_2.5_ estimates aligned within 6% of WHO-GBD assessments (Kuylenstierna *et al* 2020); in Nepal, mitigation scenarios demonstrated ±9% consistency with GEOS-Chem simulations for ΔPM_2.5_ (Nakarmi *et al* 2020); and in Ghana, avoided mortality projections showed <10% deviation compared to the GAPF health framework (Malley *et al* 2023). For Jordan, validation was conducted by benchmarking the 2016 methane and HFC baselines (MoEnv 2020a) against global inventories and IPCC-compliant reporting tools. These cross-national comparisons collectively demonstrate the fidelity of LEAP-IBC for integrated air quality–climate.

### 2.1. LEAP-IBC modeling framework

**Figure 1.**
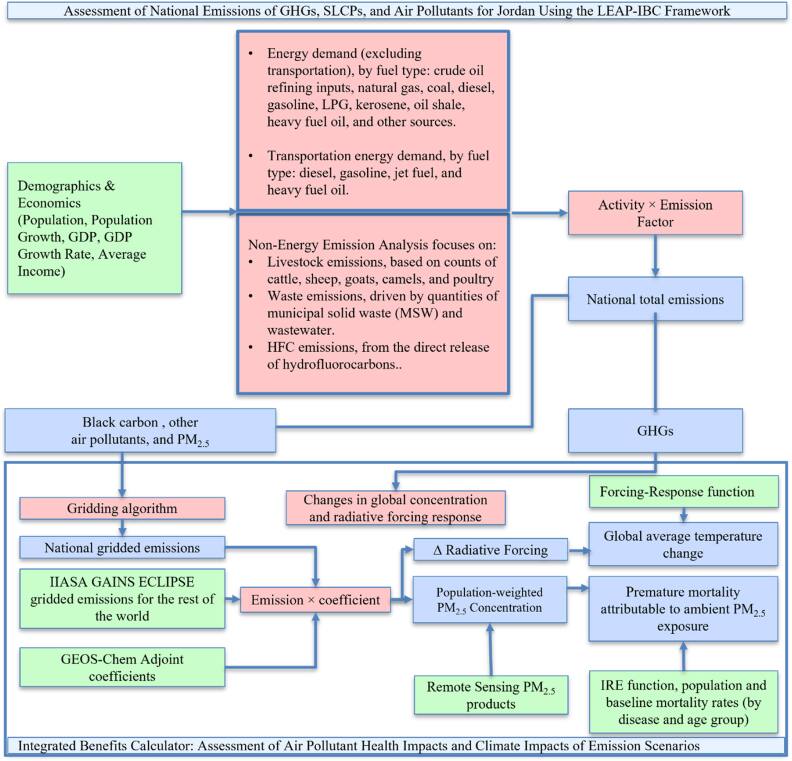
Schematic of the LEAP-IBC framework for estimating Jordan’s emission scenarios, PM_2.5_ concentrations, health impacts, and global temperature contributions. Color-coded: Inputs (green), Calculations (red), Outputs (blue).

LEAP-IBC integrates energy demand, non-energy activities, emission factors, and socio-economic drivers to simulate emissions under different scenarios (Kuylenstierna *et al* 2020). The LEAP-IBC framework (figure 1) used in this study estimates emissions of GHGs, SLCPs, and other air pollutants under both baseline and mitigation scenarios. Emissions generated by LEAP are processed by the Integrated Benefits Calculator (IBC) to assess impacts on PM_2.5_-related mortality and global temperature change. LEAP supports modeling at regional, national, and sub-national scales. A national LEAP model is particularly necessary because PM_2.5_ assessments are only available at the national scale and depend on total emissions from all major sources.

The LEAP-IBC framework transforms domestic and transboundary primary and secondary PM_2.5_ emissions into population-weighted PM_2.5_ by applying GEOS-Chem Adjoint sensitivities to quantify atmospheric transport, chemistry, and deposition. Results are downscaled using satellite-calibrated PM_2.5_ (Donkelaar *et al* 2016) to correct resolution and bias. Future PM_2.5_ projections derive from adjoint-modeled emission differences between baseline and scenario years, scaled to observed baseline concentrations. PM_2.5_ is partitioned into natural, domestic, and transboundary contributions by: (1) estimating sectoral emissions (BC, OC, NO_x_, SO_2_, NH_3_) via LEAP; (2) spatially distributing emissions (2° × 2.5° grid) integrated with GEOS-Chem adjoint sensitivities; (3) attributing natural PM_2.5_ via GEOS-Chem baseline simulations (e.g., dust, wildfires); and (4) isolating anthropogenic PM_2.5_ into domestic and transboundary fractions by subtracting natural concentrations and leveraging source-specific atmospheric transport (Kuylenstierna *et al* 2020).

In the LEAP-IBC framework, premature mortality is the primary health endpoint for assessing impacts from ambient and household PM_2.5_ exposure. Mortality attributable to PM_2.5_ is estimated for two key demographic groups: (1) children under five years, specifically for acute lower respiratory infections (ALRI); and (2) adults over 30 years, stratified into five-year age intervals as needed and assessed across multiple health endpoints (Kuylenstierna *et al* 2020).

Our exposure assessment focused on ambient PM_2.5_ as multiple portions of Jordanian populations are residing in high-ambient-pollution areas (Saadeh *et al* 2022). Relative risk (RR) of mortality is quantified using Integrated Exposure–Response (IER) functions (Burnett *et al* 2014, Cohen *et al* 2017), which integrate epidemiological data from ambient air pollution, household air pollution, and active/passive smoking. The IER function (equation (1)) used by IBC calculator is:

\begin{eqnarray*}\begin{array}{c}{\boldsymbol{R}}{{\boldsymbol{R}}}_{{\boldsymbol{IER}}}=1+\alpha \left(1-{{\boldsymbol{e}}}^{-{\boldsymbol{\gamma }}{\left({\boldsymbol{z}}-{{\boldsymbol{z}}}_{{\boldsymbol{cf}}}\right)}^{{\boldsymbol{\delta }}}}\right)\,\end{array}\end{eqnarray*}

Where z: is PM_2.5_ exposure concentration (μg/m^3^), zcf is theoretical minimum risk exposure level (μg/m^3^) and *α*,*γ*,*δ* are disease-specific coefficients derived from meta-analyses.

Premature deaths are computed for each disease, age group, year, and scenario (baseline/mitigation) using equation (2).

\begin{eqnarray*}{\mathrm{\Delta }}\begin{array}{c}Mortality={y}_{0}\left(\displaystyle \frac{R{R}_{IER}-1}{R{R}_{IER}}\right)Pop\end{array}\end{eqnarray*}

where y_0_ is the disease-specific national mortality rate (deaths per 100,000), and Pop is the size of the exposed population for each target age and disease group.

In terms of year-by-year changes in global mean surface temperature, LEAP-IBC estimates it by combining the radiative-forcing effects of both long-lived GHGs and SLCPs. Emissions are first converted to radiative forcing within four latitude bands using coefficients from Shindell (2012) framework; the resulting forcing is then translated into temperature change with pre-computed absolute regional temperature potentials, avoiding the need for new climate-model runs. This parameterization—validated against coupled chemistry-climate models—enables rapid comparison of alternative emissions scenarios while capturing the distinct warming and cooling influences of short- and long-lived species (Henze *et al* 2007).

### 2.2. Baseline emissions estimation

For the assessment of the BC emissions from Jordan, the baseline year has been set to 2022 since it was the year with the most updated sufficient and reliable data available from the relevant ministries and authorities. Additionally, it reflects the most recent representative situation following the COVID-19 pandemic. BC emissions for 2022 were calculated using the formula:

\begin{eqnarray*}\begin{array}{c}E=\displaystyle \displaystyle \sum _{j}\displaystyle \displaystyle \sum _{k}{C}_{j,k}E{F}_{j,k}\end{array}\end{eqnarray*}

*where E is total BC emissions, C*_*j,k*_ *is fuel consumption, and EF*_*j,k*_ *is the emission factor for fuel type j in sector k.*

Technology-specific emission factors (EFs) such as kerosene and solid-fuel combustion devices were not disaggregated in this national inventory due to the near-total phase-out of traditional technologies in Jordan’s residential sector. According to the Jordan Population and Family Health Survey conducted by Jordan Department of Statistics (DoS) in 2023, 99.4% of households rely exclusively on clean fuels/technologies for cooking, while only 0.2% use solid fuels. For lighting, 99.9% use clean technologies (e.g., electricity), and 78.4% use clean solutions for all energy needs (cooking, heating, and lighting combined). Consequently, kerosene wick lamps, traditional biomass stoves, and similar high-emitting devices collectively represent <0.5% of residential energy use (DoS 2024). Applying granular EFs such as 90 versus 9 g kg^−1^ for kerosene lamp types by Lam *et al* (2012) would have a negligible impact given inventory uncertainties (±30%–120%) for contained combustion (Bond *et al* 2013). Anyhow, we conservatively applied high-bound EFs from Bond *et al* (2004) to avoid underestimation. Future sub-national studies could refine this approach if localized data on heating usage become available—for example, by focusing on residential use of kerosene-based heating devices, a practice that persists by some households in Jordan.

### 2.3. Calibration and validation

Model calibration leveraged the established credibility of LEAP-IBC within Jordan’s national reporting framework as has been adopted since 2010 for the first Biennial Update Report (MoEnv 2017). We have developed our model using a previously validated profile for Jordan obtained from MoEnv and have benchmarked our 2022 baseline estimate against Jordan’s officially validated 2010 methane and HFC inventory published in the Second Biennial Update Report (MoEnv 2020a) for the baseline year 2016, that had undergone rigorous review and demonstrated alignment with IPCC methodologies and UNFCCC reporting standards. Given the absence of major policy shifts or demographic disruptions between 2016 and our baseline period, we confirmed that our emission estimates were logically consistent, residing above—but within expected variance of—the 2016 reference values. To ensure methodological continuity, adopted identical EFs for methane and HFCs to those used in the SBUR, replicated core equations for emission calculations, and conducted direct consultations with authors of the SBUR and technical staff at the MoEnv. This collaborative review formally validated our application of LEAP-IBC parameters and computational logic for the current BC inventory, providing a robust foundation for the 2022 baseline and subsequent projections.

### 2.4. Future projections

Emissions were projected to 2050 using population and GDP growth as primary drivers, with fuel consumption in the transport sector linked to population growth and energy sector consumption modeled as a function of both population and GDP growth (Sheldon *et al* 2022). Projections of fuel consumption employed the *GrowthAs* function within the LEAP-IBC environment, which calculates values based on the growth rate of a specified variable. For the transport sector, fuel consumption was explicitly linked to population growth using the following formula:

\begin{eqnarray*}{\mathrm{Fuel}}\,{\mathrm{Consumption}}\,\left({\mathrm{t}}\right)={\mathrm{Fuel}}\,{\mathrm{Consumption}}\,\left({\mathrm{t}}-1\right)\,* \,\left({\mathrm{Pop}}\,{\mathrm{Growth}}\,\left({\mathrm{t}}\right)/{\mathrm{Pop}}\,{\mathrm{Growth}}\,\left({\mathrm{t}}-1\right)\right)\end{eqnarray*}

where t represents the current year and t-1 the previous year, pop is for population.

For the energy sector, a two-step process was applied: first, GDP growth was used as the independent variable to generate initial projections, and then these values were piped into the GrowthAs function again, using population growth as an independent variable.

### 2.5. Study boundaries

Jordan’s 2022 population of 11.26 million—comprising 7.7 million Jordanians and 3.3 million non-Jordanians (including 1.3 million Syrian refugees)—exhibits extreme urbanization (91.83%, rising from 59.9% in 1980), with 42% concentrated in the country capital Amman and only 9.7% in rural areas; projected to reach 19 million by 2050 (doubling every 29 years), with its households average 4.8 persons (Macro Trends 2025). Rapid population growth, urbanization, and refugee influxes are straining energy, food, water, and infrastructure demands (MoEnv 2022a). The subsequent two paragraphs present the national current circumstances of the sectors that emit BC.

A. According to the Jordan Public Transport Diagnostic and Recommendations Report (Morad *et al* 2022), Jordan’s transport sector, dominated by road-based mobility with no rail-based transportation and a high dependence on private vehicles, while public transport accounts for only 13% of trips (5% by bus, 8% by taxi service), with 85% of public transport vehicles individually owned, leading to fragmented and inefficient services. The sector contributes 28% of Jordan’s GHG emissions (10,600 GgCO_2_-eq in 2021), projected to rise to 11,000 GgCO_2_-eq by 2030 without intervention, however, the government is advancing reforms, including the Amman Bus Rapid Transit (BRT) project, fare integration and Intelligent Transportation Systems (ITS) (Morad *et al* 2022).
B. Jordan’s energy sector provide energy for all uses (residential, industrial, agricultural, commercial etc in the form of electricity and direct fuels) and relies heavily on imported fossil fuels (92% of primary energy) (Saeeden 2011, Dar-Mousa and Makhamreh 2019, Sandri *et al* 2020). With annual energy demand rising by 3% (Abu-Rumman *et al* 2020), Jordan aims to diversify its mix via renewables (14% by 2030) (Ababneh *et al* 2023, UNDP 2023), nuclear energy (World Nuclear Association 2024), and solar potential (4–8 kWh m^−2^) (Alrwashdeh *et al* 2018), while targeting a 10% emissions reduction by 2030 (UNDP 2023). However, the rapidly growing building sector (4%–5% annually) (Nazer 2019) lacks robust decarbonization strategies, highlighting the need for housing stock models to evaluate energy performance and inform policies for reducing BC emissions (Alasmar *et al* 2024).

### 2.6. Activity data

Activity data were compiled from primary resources through formal collaboration with Jordan’s MoEnv under a national project (CCAC 2023). Data were obtained through direct correspondence with relevant Jordanian authorities, confirming that diverse fuels used in power generation and transport are major sources of BC.

In terms of data curation, fuel consumption data underwent cross-verification against the Jordan Country Balance (2022) (MEMR 2023), a validation approach previously applied in Jordan’s Fourth National Communication Report on Climate change (2022) (MoEnv 2022a) and then underwent unit conversion to kilo-tonnes of oil equivalent (ktoe). Economic and demographic data shown in table 1 were obtained from DoS (2016) and open data sources (MoEnv 2022a).

**Table 1.** Projected socioeconomic indicators (2022–2050) for Jordan.

Data	Units	2022	2030	2040	2050
Population	m People	11.345	13.45124	16.72135	20.78645
Population Growth	%/a	2	2.32909	2.895311	3.599185
GDP	USD	49.375	60.210	76.325	96.754
GDP Growth	%/a	2.4	2.83342	3.591786	4.55313
Average Income	USD/Person	4352.1375	4476.179	4564.55	4654.667

Population-standardized mortality rates (per 100,000) and population age structures for Jordan were obtained from the Institute for Health Metrics and Evaluation (IHME 2021). The dataset (2017–2021) provides detailed mortality data by age group, sex, and specific causes for six health endpoints modeled in LEAP-IBC: lung cancer, lower respiratory infections (LRIs), chronic obstructive pulmonary disease (COPD), type 2 diabetes mellitus, stroke, and ischemic heart disease (IHD), covering ages from early childhood (<5 years) to 80+ years; these data are provided in the supplementary material.

### 2.7. Emission factors and fuel data

The LEAP-IBC model estimates BC emissions by applying pollutant- and fuel-specific emission factors (EFs) to relevant activity data (Eggleston *et al* 2006). Default EFs for each country profile within the framework are provided as intended for use unless superior, region-specific or locally vetted EFs are available. The primary sources for these BC emission factors are: (a) the Tier 1 default EFs in the IPCC 2006 Guidelines (Eggleston *et al* 2006); (b) the EMEP/EEA (2016) Tier 1 emission factors (EEA 2016); and (c) central values from Bond *et al* (2004) who assume that 52% of gasoline vehicles are ‘unimproved’, 35% are 2-stroke (high emissions) and 13% are ‘super-emitters’. Researchers, particularly in East Asia including Jordan—predominantly employ the EFs ranges outlined by the IPCC, EMEP/EEA, and Bond *et al* (2004) due to the scarcity of region-specific EF measurements, which impedes justification for deviating from these established default sources.

It should be noted that EFs may vary significantly with technology and fuel composition, e.g., kersone lamps and regional sulfur content, as demonstrated by Lam *et al* (2012) - yet Jordan’s 2022 National Energy Balance (MEMR 2023)— our primary data source — lacks the fuel-grade and technology detail needed to explore such distinctions. Jordan’s energy supply is primarily processed by the Jordan Petroleum Refinery Company (JPRC), which refines imported crude oil into standardized petroleum fuels—including unleaded gasoline-90 (minimum PFOA 90), gasoline-98 (minimum PFOA 98), diesel (normal and special grades), kerosene (JS 194:2017), jet fuels (Jet A-1 civilian and JP-8 military), heavy fuel oil, and liquefied petroleum gas (LPG, JS 298/2024)—in addition to direct imports of refined products and natural gas to meet national demand (Jordan Petroleum Refinery 2025). Consequently, the absence of technology-specific or fuel-composition-adjusted EFs is unlikely to substantially affect our results, and we therefore rely on the EFs identified in table 2, with the caveat that future national inventories should incorporate enhanced monitoring of fuel characteristics and technology use. EFs will be assumed steady as the changes in BC EFs over time are relatively modest (Klimont *et al* 2017), as improved technology usually is more effective at reducing overall particulate matter emissions rather than BC.

**Table 2.** Sectoral black carbon (BC) emission factors (EFs) by fuel type, showing fuel consumption (in kilotonnes of oil equivalent, ktoe), applied EF values (kg BC per tonne of fuel), and literature-reported EF ranges.

Sector	Fuel type	Fuel consumption (ktoe)^^a^^	EF used (kg/t)^^b^^	Source of EF value	Range of reported BC EFs (kg/t)
Energy	Coal	225.8	0.83	Bond *et al* (2004)	0.002–0.009 (Power)^^c^^
					0.013–1.2 (Industry)^^c^^
					0.76–5.4 (Residential)^^c^^
	Diesel	508.2	3.6	Bond *et al* (2004)^^d^^	0.06–4.0 (Residential)
					3.4–4.4 (Industry)
					0.25 (power)
	Gasoline	0.2	0.43	Bond *et al* (2004)	0.14–0.9
	Kerosene	72.5	0.45	EMEP/EEA (2016); Tier 1 (1.A.4, Table 3.9)	0.14 (Industry)^^c^^
					0.9 (Residential)^^c^^
	LPG	599.3	0.1	Bond *et al* (2004)	0–0.2 (Residential)
	Natural Gas	3,443.4	0.002	Bond *et al* (2004), EMEP/EEA (2016); Tier 1 small combustion (1.A.4, Table 3.8)	0–0.03
	Heavy Fuel Oil	1630	0.05	Bond *et al* (2004)	0.04 (Power & Industry)
					0.07 (Residential)
	Other Fuels^e^	307	0.45	EMEP/EEA (2016); Tier 1 (Table 3.9)	0.14–0.90
Transportation	Diesel	1,151.6	3.6	Bond *et al* (2004)^^f^^	0.34 (Ships)^^c^^
					1.3–3.6 (Vehicles)^^c^^
					2.6–3.7 (Tractors)^^c^^
	Fuel Oil	4.2	0.27	EMEP/EEA (2016); Tier 1 ocean-going ships (1.A.3, Table 3.5)	0.0528–0.442
					0.34 (ships)
	Gasoline	1,459.8	0.43	Bond *et al* (2004)	0.08–0.43
	Jet Fuel	307.7	0.1	Bond *et al* (2004)	0.1

^a^ Consumption in thousand tonnes of oil equivalent (ktoe), sourced from Jordan’s Ministry of Energy and Mineral, 2023

^b^ EFs are the values implemented in the model

^c^ Sub-sector specific ranges reported in literature for uncertainty analysis

^d^ Based on mid-1990s Indian technology/control mix.

^e^ Unspecified minor fuel streams aggregated under ‘Energy’

^f^ EF value applies to road vehicles; see range column for other transport modes (Eggleston *et al* 2006).

aggre

### 2.8. BC emissions conversion into CO_2_ equivalent

Converting BC emissions to CO_2_-equivalent (CO_2_-eq) involves using the Global Warming Potential (GWP), which compares the warming impact of a greenhouse gas to that of CO_2_ over a specific time horizon (typically 20 or 100 years), however, BC does not have a globally standardized GWP like methane as an example (EPA US 2025b). Nevertheless, BC exhibits much higher GWPs than CO_2_ (UNEP and WMO 2011) and the choice of time horizon for calculating impacts involves a trade-off: longer horizons emphasize long-term climate stabilization, whereas shorter horizons capture intense short-term warming (IPCC 2014). In this study, we modeled the range of GWPs reported in table 3, drawing partly from IPCC AR5 (Chapter 8, p. 740) and from values proposed by other research groups. This approach allowed us to estimate the range of BC emissions in CO_2_-eq and to capture the associated uncertainty (IPCC 2014).

**Table 3.** Comparison of published global warming potential (GWP) values for black carbon (BC) across key studies, showing time horizons (20- and 100-year), reported values (mean ± uncertainty or range), and scope of climate effects considered.

Source	Time horizon (years)	GWP value (mean ± uncertainty or range)	Scope of effects included	Notes
(Collins *et al* 2013)^a^	100	345 ± 207	Aerosol-Radiation Interaction (ARI) only	Most conservative value: recommended for illustrative CO_2_-eq metrics and excludes indirect effects; lower than total GWPs
	20	1,200 ± 720		
(Bond *et al* 2013)	100	900 (100–1,700)	Total effect (ARI + aerosol-cloud interactions + snow/ice albedo + semi-direct effects)	Represents full BC warming impact
	20	3,200 (270–6,200)		
(Fuglestvedt *et al* 2010)	100	460	Aerosol-Radiation Interaction (ARI) only	Global level
	20	1,600		
(Bond and Sun, 2005)	100	680	Aerosol-Radiation Interaction (ARI) only	Direct forcing only; excludes indirect effects (cloud interactions, snow albedo).
	20	2,200	Aerosol-Radiation Interaction (ARI) only	Short-term focus; direct forcing only. Higher uncertainty due to atmospheric lifetime variability.
Ministry of Environment^b^	100	345	Aerosol-Radiation Interaction (ARI) only	Adopted for national climate tracking
	20	1,200		

^a^ regional analysis (East Asia, EU + North Africa, North America)

^b^ formally adopted value for national reporting (Henze *et al* 2007).

For official national climate reporting to the MoEnv, a conservative approach to GWP quantification has been adopted, utilizing values of 345 (100-year horizon) and 1,200 (20-year horizon). These values correspond to the Aerosol-Radiation Interactions (ARI)-only regional averages reported by Collins *et al* (2013), excluding indirect effects such as Aerosol-Cloud Interactions (ACI) and albedo contributions. This methodology aligns with current national inventory systems of other gases and emphasizes conservatism in climate risk assessment. However, this approach warrants critical evaluation given BC’s short atmospheric lifetime and its disproportionate impact on near-term climate forcing. Bond and Sun (2005) estimate a 20-year GWP of 2,200 for BC—approximately twice higher than the adopted conservative value—reflecting inclusion of total climate effects beyond ARI. To address these scientific and policy imperatives, we have done dual horizon reporting. The equation for calculating CO_2_ equivalent emissions as programmed by LEAP is:

\begin{eqnarray*}{{\mathrm{CO}}}_{2}-{\mathrm{eq}}\,\left({\mathrm{tons}}\right)={\mathrm{BC}}\,\left({\mathrm{tons}}\right)\times {\mathrm{GWP}}\end{eqnarray*}

### 2.9. Mitigation measures identification and stakeholder engagement

Mitigation measures targeting BC reduction were identified through expert input and review of national policy documents, including Jordan’s NDCs, National Communications, Climate Policy, Biennial Reports, and sectoral ministry strategies. These measures were categorized into short-term (2022–2029), mid-term (2030–2039), and long-term (2040–2050) implementation phases, and subsequently validated through focus group discussions (FGDs) with more than 40 representatives from key ministries and agencies (e.g., Energy, Transport, LTRC, CARC, Environment). The FGDs assessed feasibility, prioritized interventions based on implementation barriers and estimated fuel-saving potential and informed the development of four LEAP-IBC scenarios: a Baseline Scenario (business-as-usual emissions) and three Mitigation Scenarios incorporating the prioritized short-, mid-, and long-term measures.

## 3. Results and discussion

### 3.1. BC emissions baseline and projections

**Figure 2.**
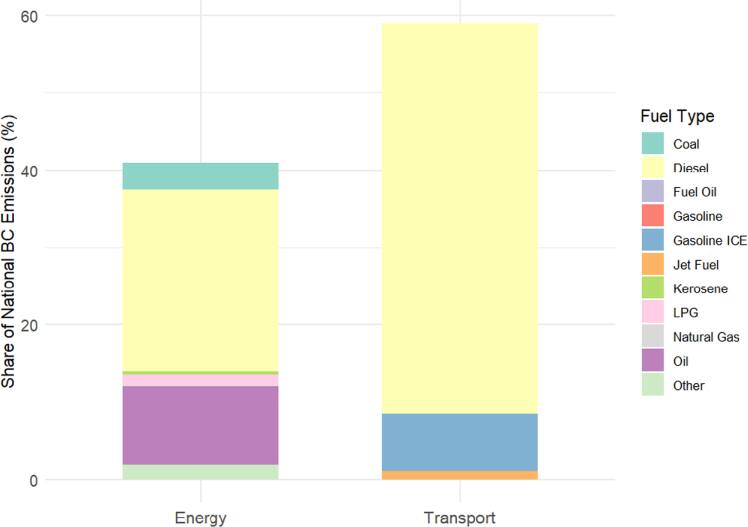
Black Carbon (BC) by sector and fuel type (proportional to national total), with bar heights reflects sector share; and segments reflect fuel breakdown within each sector in Jordan.

Black carbon (BC), a significant short-lived climate pollutant (SLCP) in Jordan, exhibited 2022 emissions of 6,977 tonnes (95% uncertainty range: 2,582–7,460 tonnes). The quantified uncertainty range in BC emission estimates arising from Emission Factors (EFs) variability, with a systematic analysis was implemented by first characterizing EFs uncertainty sources through compilation of literature-derived ranges (see table 2), addressing aleatory uncertainty (inherent technological variability) and epistemic uncertainty (knowledge gaps in local conditions); this was followed by quantitative uncertainty propagation using sensitivity analysis, where sectoral BC emissions were calculated separately with minimum (50% less than those vetted for Jordan, vetted values, and maximum EFs) to determine emission ranges, establishing bounds for potential estimation error. In 2022, BC emissions in Jordan were distributed between the transport sector (59% ± 8%) and the energy sector, which accounted for the remainder (figure 2). Within transport, diesel and gasoline combustion dominate, while in the energy sector, oil combustion and diesel-based power generation are the primary contributors—underscoring the urgent need for sector-specific mitigation measures such as fuel transitions to cleaner alternatives and enhanced industrial efficiency.

**Figure 3.**
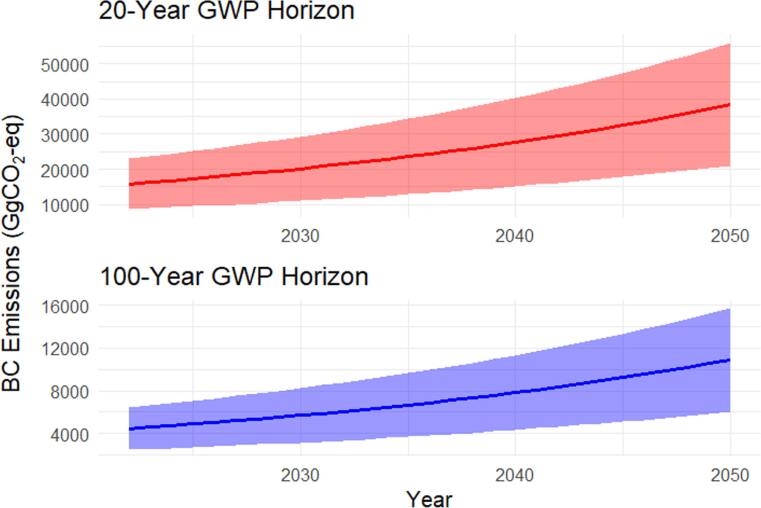
Dual-panel uncertainty band plot of BC emissions (GgCO_2_-eq) from 2022 to 2050 under the business-as-usual scenario under 20-year and 100-year GWP horizons, showing minimum, maximum, and mean trajectories based on GWP value ranges.

Figure 3 compares BC emission trajectories in Jordan from 2022 to 2050, reported in GgCO_2_-eq. National BC emissions are shaped by two structurally distinct sources of uncertainty embedded in the GWP metric: (i) the time horizon (20-yr versus 100-yr) and (ii) the treatment of forcing pathways (high versus low estimates, here labeled ‘max’ and ‘min’). For 2022, CO_2_-eq emissions vary widely across GWP frameworks, ranging from 2,480–23,004 GgCO_2_-eq: the 20-year horizon yields 23,004 GgCO_2_-eq using GWP = 3,200 (total radiative effect) and 8,626 GgCO_2_-eq using GWP = 1,200 (direct effect only), whereas the 100-year horizon produces 6,470 GgCO_2_-eq at GWP = 900 (total effect) and 2,480 GgCO_2_-eq at GWP = 345 (direct effect) (Bond *et al* 2013, Collins *et al* 2013). By 2050, BC emissions span a wide range of 6,034–55,970 GgCO_2_-eq using both 20 and 100 years GWP, with GWP_100_ producing lower estimates (6,034–15,742 GgCO_2_-eq) due to long-term dilution. This metric-driven variability underscores the dominant role of GWP selection in shaping uncertainty in BC climate impact assessments.

### 3.2. Cross-national comparison of emission estimates

Global emission inventories have long provided estimates of BC emissions for all countries. The Emissions Database for Global Atmospheric Research (EDGAR) compiles BC and other air pollutants by country and broad sector categories (e.g. power, industry, transport, buildings) in consistent time series. EDGAR’s dataset (e.g. v8.1, derived from EDGARv4.3.2/5.0) spans 1970–2022 and is built on international activity data (primarily IEA energy statistics and FAO agricultural data). Notably, EDGAR focuses on anthropogenic sources and excludes large-scale biomass burning (wildfires, savannah fires, and other LULUCF sources) (EDGAR 2022). Similarly, the Community Emissions Data System (CEDS) provides standardized annual BC emissions from 1750 up to recent years, with data reported by country and sector. CEDS uses a consistent methodology that harmonizes energy use data and regional inventories to produce national trends. In practice, CEDS estimates for BC are often slightly higher than other global inventories and it acknowledges larger uncertainties for regions lacking dedicated local inventories (Hoesly and Smith 2024). However, these global datasets are based on broad assumptions and default EFs. As a result, their estimates for a given country can diverge and may not capture country-specific conditions. For instance, global inventories do not always reflect diffuse, inefficient combustion sources prevalent in developing regions—a known limitation that can lead to under- or over-estimation of BC. EDGAR and CEDS inventories appear to systematically underestimate BC emissions for Jordan and other countries relative to national inventories, a pattern that is consistent across multiple cases (table 4). These discrepancies suggest that global inventories employ conservative EFs reflecting optimistic BC abatement conditions. Sun *et al* (2019) showed that earlier U.S. BC inventories substantially underestimated emissions because EFs for pre-regulation vehicles and pre-1980 residential stoves/boilers were set too low, and they recommend raising these factors by 70%–250%, which increased U.S. 1960 BC emissions to about 690,000 tonnes (excluding open burning), and reveals a strong downward trend driven by subsequent regulatory controls. Such methodological limitations highlight substantial uncertainties in BC emission modeling and underscore the necessity of country-specific validation frameworks.

**Table 4.** Comparison of national black carbon (BC) emission estimates (in tonnes) across different inventories for selected countries. Values are shown for the reference years specified as sourced from EDGAR v8.1 (2024), CEDS (2021) and national initiatives.

Country	Reference Year	National Initiatives	LEAP-IBC framework	EDGAR v8.1	CEDS (2021)
**Jordan (this study)**	2022	2,582–7,460	Yes	1,007.02	1,567.81
**Bangladesh** (Kuylenstierna *et al* 2020)	2010	50,290	Yes	27,303.70	39,992.17
**Ghana** (Ghana Environmental Protection Agency 2018)	2010	15,900	Yes	12,211.61	10,409.28
**Nepal** (Nakarmi *et al* 2020)	2015	39,626	Yes	21,189.37	35,836.48
**Dominican Republic** (Ministry of Environment of Dominican Republic 2021)	2010	4,067.5	Yes	4,892.62	5,045.06
**USA** (EPA US 2025a)	2022	406,000	No	195,877.74	126661.7
**Russia** (Evans *et al* 2017)	2014	688,000	No	46,449.99	175,023.3
		(401000–1,453000)			
**China** (Wang *et al* 2012)	2007	1,957,000	No	1,306, 816.46	1,707,958.00
**India** (Paliwal *et al* 2016)	2011	901,110	No	635323.28	797470

In developing BC emission inventories, bottom-up methods, as the LEAP-IBC framework used in our study, remain foundational, combining activity data with EFs to estimate sources. Another example, an updated inventory for China (1949–2007) introduced improved EFs and high-resolution (0.1°) gridding, revealing higher BC emissions than previously reported and identifying residential coal as a top source (Wang *et al* 2012). Such inventories are policy-relevant but can underestimate true emissions if local factors are off—one study found that late-20th-century U.S. BC emissions were ∼80% higher than earlier inventories suggested after considering discrepancies between observations and predicted emissions among several sources based on seasonal and weekly patterns in field observations (Sun *et al* 2019)**.** Ther are also Top-down, observation-driven methods combine satellite and surface measurements to directly infer BC emissions, for instance, Liu *et al* (2025) used satellites to directly estimate BC emissions in Asia and found that real emissions are much higher than traditional datasets because many urban and fire sources were missing or underestimated. By comparing mass- and number-based methods, they showed that particle counts matter a lot for getting emissions right. Hybrid approaches integrate multiple datasets and modeling strategies. For example, Jin *et al* (2024) tested eight different fire emission datasets within the same climate model to evaluate smoke and climate impacts over Southeast Asia in March 2019. They then compared the model outputs with satellite and ground-based observations, enabling them to identify which inventories produced the most realistic results. Evans *et al* (2017) demonstrated that Lagrangian receptor models can trace measured pollution back to probable source regions, enabling the attribution of acute BC events to biomass burning and other upwind sources. A receptor model begins at the measurement site and traces air parcels backward in time to identify the regions or activities that contributed to the observed pollution, rather than simulating emissions forward from sources. However, their quantitative reliability decreases with increasing transport time due to compounding meteorological uncertainties. Despite this limitation, such models remain valuable for improving emission datasets and refining source attribution. Together, these bottom-up, top-down, hybrid, and receptor-based approaches highlight both the progress and persistent uncertainties in BC emission estimation, underscoring the need for integrated methods that leverage observational constraints to refine inventories and better inform air quality and climate policy.

### 3.3. Impact on climate

Jordan’s cumulative emissions have contributed approximately 0.00052 °C to global warming, accounting for just 0.04% of the total 1.3 °C rise since pre-industrial times by 2021 (Jones *et al* 2023). Our study estimates that projected emissions from Jordan between 2022 (baseline) and 2050 are expected to cause a net global mean surface temperature increase of 0.000150 °C, dominated by positive radiative forcing from CO_2_ (+0.000099 °C, 66.0% of total change) and BC aerosols (+0.000058 °C, 38.7%). This warming is partially offset by net cooling effects from aerosols such as sulfate (SO_x_; −0.000027 °C) and organic carbon, which collectively contribute a net reduction of −0.000007 °C (4.7% of total change). BC exhibits the most rapid growth trajectory, escalating to be the second-largest warming agent by 2050, reflecting increased emissions or insufficient mitigation.

### 3.4. BC health burden

**Figure 4.**
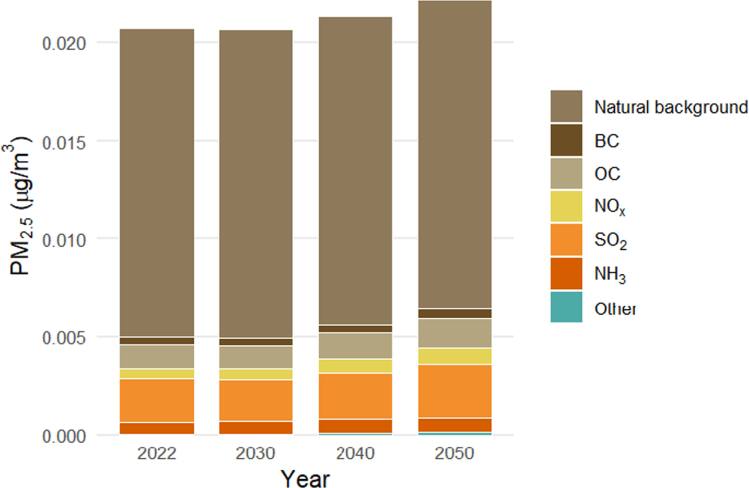
PM_2.5_ population-weighted annual average across Jordan baseline projections disaggregated by contribution from different pollutant emissions within the country (National) and outside the country (other), and from natural background.

In Jordan, the population-weighted annual PM_2.5_ concentration was estimated at 20.72 ± 0.015 μg m^−3^ in the baseline year 2022, composed of 15.76 ± 0.0114 μg m^−3^ from natural background sources (e.g., dust storms) and 5.00 ± 0.0036 μg m^−3^ from anthropogenic emissions. By 2050, PM_2.5_ levels are projected to rise slightly to 22.19 ± 0.002 μg m^−3^ (See figure 4). Source apportionment shows that 75% of PM_2.5_ originates from natural background sources, 20% from transboundary anthropogenic emissions, and 5% from national emissions. BC accounted for 1.7% of total PM_2.5_ in 2022, with organic carbon (OC) contributing 6%, of which 0.7% stemmed from national emissions (e.g., transport and energy sectors), while 1.0% originated from international sources. By 2050, BC’s contribution to PM_2.5_ is projected to nearly double to 3.3%, with national emissions’ share rising to 1.3%.

PM_2.5_-attributable deaths with our age-distribution analysis reveals over 60% of premature deaths occurred in adults over 70 years old, while children under five accounted for <2% of the total burden. Premature mortality is projected to increase substantially to 14,300 ± 34 deaths by 2050 under baseline scenario. This surge stems not only from rising PM_2.5_ concentrations but also from demographic shifts. Jordan’s population is forecast to grow from ∼11 million (2022) to 21 million (2050), amplifying exposure. The age distribution used in LEAP focuses on under-five children and adults over 30, shows that 71.7% of female and 52.4% of male premature deaths occur in the >70 age group, with another 21%–35% in the 50–70 age group. Between 2022 and 2050, the proportion of Jordanians over 70 will double, Consequently, a larger elderly population will face elevated PM_2.5_ exposure, disproportionately driving future mortality burdens given their established vulnerability.

**Figure 5.**
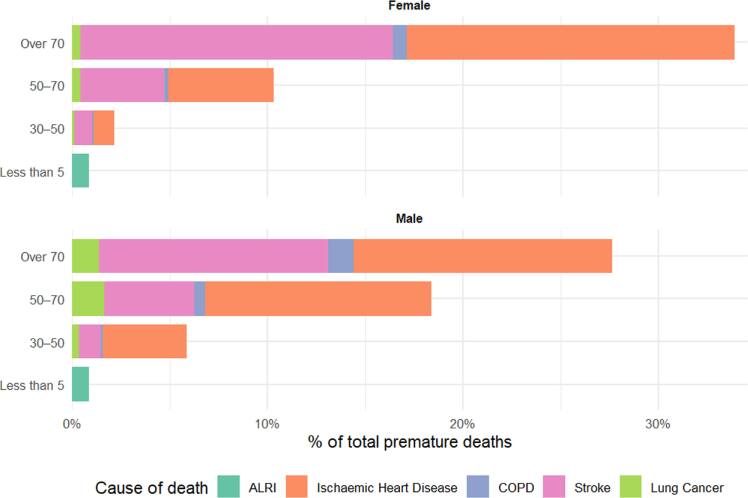
Relative contribution to PM_2.5_-attributable premature deaths, Jordan 2022. Percent of estimated national total by gender, age group, and disease.

In terms of leading causes of death (figure 5), cardiovascular and respiratory diseases dominate with Ischaemic heart disease (IHD) accounts to about 52.3% of total attributable mortality, followed by stroke (38.6%), collectively representing >90% of the burden while lung cancer and COPD contributed modestly (4.4% and 2.9%, respectively), while acute lower respiratory infections (ALRI) exclusively drove mortality in children under five (1.8%, 100% of group burden). Significant demographic disparities were identified, e.g. IHD exhibited a pronounced male predominance (peaking in ages 30–70), whereas stroke disproportionately affected females >70 years. Lung cancer mortality more in males (77% of cases), particularly in the 50–70 age group. Among adults 30–50 years, cardiovascular diseases dominated (92.2% of deaths), with IHD comprising 66.7% of deaths (86.65) and exhibiting extreme male predominance (80% of IHD cases). This pattern intensified in adults 50–70 years, where IHD accounted for 59.1% of deaths (273.59), while lung cancer burden surged to 7.3% (33.96 deaths) with 79% male vulnerability. Critically, adults >70 years bore 61.5% of all PM_2.5_-attributable deaths with IHD remained primary (48.7%) but with female predominance (56%), while stroke represented 45.0% of deaths (446.86) with 58% female vulnerability, underscoring extreme elderly vulnerability to air pollution-driven cardiovascular outcomes. These data demonstrate an epidemiologic transition: from purely respiratory mortality in early childhood, to working-age cardiovascular disease with pronounced male susceptibility, culminating in elderly cardiovascular burden with sex-specific organ vulnerability (female stroke versus male COPD).

Between 2022 and 2050, population-weighted mean BC exposure is projected to increase from 0.35 μg m^−3^ to 1.65 μg m^−3^. In 2022, BC exposure was linked to about 30 excess premature deaths (out of about 1,600 ± 14 total PM_2.5_-attributable deaths). By 2050, annual BC-related premature deaths are projected to reach about 340, highlighting their increasing role in air pollution’s health impacts.

### 3.5. Scenario-based evaluation of black carbon abatement strategies

In addition to the Business-as-Usual (BAU) baseline, three mitigation pathways—short-, mid-, and long-term—were formulated based on consensus-identified climate measures in the transport and energy sectors. Candidate interventions with demonstrable BC abatement potential were screened, ranked by implementation feasibility, and quantified for expected fuel-consumption savings through a systematic assessment framework. The resulting portfolio was synthesized from ministerial stakeholder consultations, sectoral roadmaps, and key national instruments—including Jordan’s NDCs, National Communications, Climate Policy, and Biennial Update Reports—supplemented by expert elicitation. Table 5 summarizes the prioritized mitigation measures, grouped by sector (transport, energy) and implementation horizon (short-, mid-, long-term). For each action, projected BC abatement is reported in annual GgCO_2_-eq, using the 100-year GWP of 345, with short-term measures defined relative to the 2022 baseline year adopted in the modeling.

**Table 5.** Mitigation actions and their estimated black-carbon abatement potential to 2050 (expressed as GgCO_2_-eq/yr, 100-year GWP = 345).

Sector	Term	Mitigation measure	Δ BC (GgCO_2_-eq/yr)
Transportation	Short-term	Amman Bus Project (Phases I & II)	28.81
		Electric buses in Amman	0.50
		Expansion of existing Intelligent Transportation System (ITS)	51.67
		Promotion of hybrid & electric vehicles	0.50
		Increased public-transport ridership	56.36
		Ring-road development	50.56
		Solar-powered electric-bus fleet	72.33
		Computerised transport-forecasting system	50.56
		New ITS deployments	53.97
		Modernisation of public-transport bus fleet	35.59
		**Subtotal (short-term)**	**400.85**
	Mid-term	Gradual ITS expansion	65.51
		Continued bus-fleet modernization	32.76
		Carbon taxes on vehicles	0.01
		Transition to alternative fuels	65.51
		Bus Rapid Transit, Phase 2 (43 km)	65.51
		**Subtotal (mid-term)**	**229.29**
	Long-term	Ongoing bus-fleet modernization	64.63
		Amman–Salt sustainable mass-transit system (feasibility)	64.63
		Low-carbon freight via rail	32.31
		**Subtotal (long-term)**	**161.57**
		**Total transportation sector**	**791.71**
Energy	Short-term	Industrial energy-efficiency upgrades	599.89
		Grid-integration infrastructure rehabilitation	299.95
		Accredited clean-energy training for engineers	299.95
		Zero-emission-vehicle (ZEV) incentives	119.98
		Solar-powered charging-station rollout	30.00
		**Subtotal (short-term)**	**1 349.77**
	Long-term	Raise renewables share to 20 % (by 2050)	264.68
		Large-scale transition to electric vehicles (residential level)	364.64
		Deployment of hydrogen energy	145.86
		**Subtotal (long-term)**	**775.18**
		**Total energy sector**	**2 124.95**
—	—	**Grand total (all measures) by 2050**	**2 916.66**

The short-term scenario focuses on transforming Jordan’s public transport system to reduce emissions and improve efficiency. Key initiatives include the Amman Bus Project, which has introduced over 250 EURO-5 fuel buses since 2018, with plans to expand to 335 buses by the end of 2024, including the Amman-Zarqa line. Additionally, 16 electric buses will be tested on Bus Rapid Transit (BRT) lines in 2025, supported by financial incentives, charging infrastructure, and public awareness campaigns to promote hybrid and electric vehicles. The Intelligent Transportation System (ITS) is being expanded with 350 new devices to enhance bus management and reduce emissions, while ring road developments aim to alleviate congestion. Efforts also include modernizing the bus fleet, studying solar-powered electric buses, and implementing a computerized transport forecasting system using VISUM (PTV group 2025), a world’s leading transport planning software, to simulate traffic impacts. These measures collectively aim to reduce fuel consumption, lower emissions, and encourage public transport usage, contributing to a cleaner and more efficient transport network. The short-term scenario in the energy sector focuses on efficiency improvements, infrastructure upgrades, and capacity building. Key measures include modernizing industrial energy efficiency to reduce BC and methane emissions, upgrading the national grid to support over 3,000 electric vehicle charging stations, and providing accredited training for engineers on clean energy technologies. Additionally, the Zero-Emission Electric Vehicles (ZEV) Program aims to deploy 10,000 renewable-energy-powered electric vehicles, while solar-powered charging stations will further promote sustainable transportation. These initiatives are projected to significantly cut emissions, with combined reductions of 983.56 GgCO_2_-eq from industrial efficiency, 491.78 GgCO_2_-eq from grid upgrades and training, 196.72 GgCO_2_-eq from the ZEV program, and 49.18 GgCO_2_-eq from solar-powered charging stations.

The mid-term scenario focuses on further enhancing Jordan’s transport system through advanced technologies and sustainable practices. Key initiatives include the continuing gradual implementation of ITS, Integrating Information and Communication Technology (ICT) solutions like Closed-Circuit Television (CCTV) monitoring, electronic tracking, and payment systems to reduce congestion and improve travel efficiency. The modernization of the public transport bus fleet continues, replacing older public buses with more efficient models to cut emissions. A regulatory framework for carbon taxes on private vehicles is being developed to incentivize lower emissions. Additionally, the transition to alternative fuels in maritime and aviation sectors aligns with international standards set by the International Maritime Organization (IMO) and the International Civil Aviation Organization (ICAO). The BRT network will expand with a 43 km Phase 2 project, connecting South and North Amman, linking to Phase I, and providing direct services to Madaba City and Queen Alia Airport.

The long-term scenario emphasizes sustainable and efficient transport solutions for Jordan. It includes the continued modernization of the public transport bus fleet, replacing older public buses with newer, more efficient models to reduce emissions, a process ongoing since 2008. Feasibility studies are being conducted for an ‘Organized and Sustainable Mass Transit System’ along the Amman-Salt corridor, aiming to define optimal public transport solutions, analyze feeder systems, and provide financial recommendations to the government. Additionally, the promotion of low-carbon freight transport via rail is a priority, with plans to transition to electric freight systems and introduce rail infrastructure. Key projects include the Aqaba Potash Railway Line (196 km) transporting potash from mines to the industrial seaport at Aqaba. These initiatives aim to create a sustainable, low-emission transport network for both passengers and freight. The long-term scenario in the energy sector focuses on transformative changes to achieve deep decarbonization and reduce reliance on fossil fuels. Key actions include increasing the share of renewable energy by an additional 20% by 2050, projected to reduce emissions by 464.27 GgCO_2_-eq. The transition to electric vehicles, targeting a 50% adoption rate by 2050, is expected to cut emissions by 597.85 GgCO_2_-eq. Additionally, investments in hydrogen energy infrastructure for production, storage, and distribution aim to reduce emissions by 239.14 GgCO_2_-eq. Together, these measures will drive structural shifts in the energy sector.

**Figure 6.**
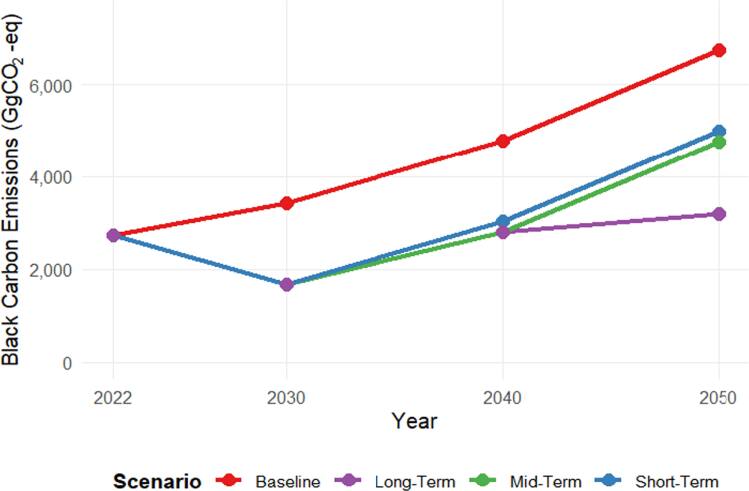
The 100-GWP of black carbon emissions under different mitigation scenarios for Jordan.

Figure 6 compares BC emissions, expressed using the 100-year GWP of 345, across four pathways—Baseline, Short-, Mid-, and Long-Term. All mitigation trajectories achieve an identical ∼39% reduction from the 2022 reference level by 2030, but they diverge thereafter. The Short-Term pathway plateaus, while the Long-Term pathway continues to reduce emissions, ultimately achieving a 53% reduction relative to BAU and remaining 36% lower than the Short-Term case by 2050. These results highlight the cumulative benefits of sustained policy, technological, and behavioral interventions.

## 4. Black carbon in Jordan: health, climate, and policy imperatives

Despite progress in renewable energy adoption and transport modernization, BC emissions driven by fossil fuel dependence continue to undermine these gains. Diesel combustion accounts for 67% of national BC emissions, a legacy of Jordan’s reliance on imported petroleum and delayed transitions to low-carbon infrastructure (Wang *et al* 2023). These emissions are compounded by demographic and economic pressures, which collectively drive a projected tripling of BC emissions by 2050 (The World Bank 2020). This trajectory threatens Jordan’s climate goals and highlights the regional tension between development and sustainability.

Jordan’s estimated population-weighted annual average PM_2.5_ concentration of 20.76 μg m^−3^ substantially exceeds the WHO guideline of 5 μg m^−3^ (WHO 2021). Although BC contributes only about 1.7%, it exerts disproportionate health and climate impacts (Hussein *et al* 2019). Mitigating BC could yield immediate health co-benefits, particularly in urban centers like Amman, where traffic-derived PM_2.5_ exceeds 35 μg m^−3^ (Hussein *et al* 2019). However, Jordan’s climate policies prioritize CO_2_ reduction while neglecting BC, reflecting a broader challenge where short-term economic growth often eclipses long-term sustainability (SEI *et al* 2023). For instance, ongoing investments in oil shale, such as the Attarat Power Plant, contradict renewable energy targets and exacerbating BC emissions make the energy sector contributing to BC emissions (IRENA 2021).

The transboundary nature of BC further complicates the issue with, 20% of its PM_2.5_ originates from cross-border pollution, necessitating regional cooperation under frameworks like the United Nations Convention to Combat Desertification (UNCCD 2022). This dual dynamic—local emissions with global consequences and imported pollution with local health impacts—demands a holistic approach to air quality and climate governance.

Thus, Jordan must adopt a dual-strategy approach. Immediate actions should include enforcing Euro VI standards for diesel vehicles, which could reduce BC emissions by up to 90% or more, as advanced diesel particulate filters (DPFs) in Euro VI-compliant vehicles are highly effective at capturing particulate matter, including BC (UNEP and WMO 2011). The current transportation fleet in Jordan, is predominantly composed of older and high-emitting technologies. As indicated by 2022 data (found in the supplementary data), the vast majority of buses—4,067 out of 4,075—still rely on diesel fuel, with only one electric bus registered, and just 135 buses reportedly using Euro V diesel engines for the BRT system. Similarly, a significant number of other vehicle categories such as shared transportation (174,602 diesel), small rides (271 diesel), and construction vehicles (19,609 diesel) remain dependent on conventional diesel engines, many of which likely fall below Euro III standards due to inadequate fuel quality and outdated emissions enforcement. According to a the Transportation Sector Green Growth National Action Plan 2021–2025 (MoEnv 2020b), Jordan’s heavy-duty vehicles largely operate under the equivalent of Euro III standards, which include diesel sulfur content at 50 ppm—far below best practices globally. Furthermore, while emission limits for CO, CO_2_, and hydrocarbons exist, enforcement mechanisms remain weak, and vehicle inspections are not systematically nor rigorously applied. Super-emitters, particularly those emitting dense black smoke, likely remain undetected or unpenalized, despite the legal provision of a 700 USD fine. This reality significantly undermines the effectiveness of the regulatory framework.

This study supports a transition toward Euro VI standards, particularly for new fleet purchases and policy planning, noting that while Euro VI fuels are available at some stations, demand remains low and cleaner technologies are underutilized. Our analysis did not account for fleet heterogeneity or disaggregate BC contributions by vehicle type, engine age, or emission standard due to limited data, representing a critical research gap given the predominance of diesel vehicles and low-grade fuels. Future studies should adopt a tiered emission factor approach, assigning BC emission rates by vehicle class, fuel type, and standard (e.g., Euro III–V), drawing on established databases such as the EMEP/EEA Guidebook and ICCT. This would also help highlight the disproportionate impacts of high-mileage diesel fleets (e.g., shared transport, construction vehicles) and the absence of particulate filters or inspection systems.

Additionally, accelerating the electrification of public transport through initiatives like the Amman Bus Project would further reduce emissions and contribute to cleaner air. Systemic reforms are equally critical, such as integrating BC-specific targets into revised NDCs and leveraging BC’s high GWPs to unlock climate finance for solar-powered charging infrastructure and desertification control programs (IPCC 2021).

Natural sources dominate PM_2.5_ exposure (∼75%), largely from desertification. Dust storms, though often considered unavoidable, are intensified by land degradation from overgrazing, deforestation, and unsustainable farming (FAO and UNEP 2020). Regenerative land management—such as afforestation and soil stabilization—could curb windblown dust while strengthening ecosystem resilience. At the same time, BC mitigation in transport (e.g., electric mobility) and energy (e.g., solar grids, green hydrogen) would reduce anthropogenic PM_2.5_ and limit BC’s climate forcing. Integrating desertification control with sectoral BC policies offers a unified pathway for air quality and climate resilience (Liu *et al* 2013).

Despite these opportunities, the current mitigation efforts fall short. Existing GHG measures could achieve if all implemented perfectly up to 53% BC reduction by 2050, but BC remains a byproduct, not a priority. The transportation sector’s reduction potential hinges on scaling electric vehicles and intelligent transport systems, yet these face barriers such as limited charging infrastructure, subsidy dependencies, and behavioral inertia (Sheldon *et al* 2022). Similarly, the energy sector’s reduction relies on accelerating renewables adoption, yet oil shale investments signal conflicting priorities (IRENA 2021). To break this impasse, Jordan must adopt a BC-Specific Mitigation Framework, incorporating policy reforms (e.g., codifying BC into NDCs), technological advancements (e.g., AI-driven emission monitoring), and equity-focused interventions (e.g., targeting low-income urban corridors where PM_2.5_-BC synergies exacerbate respiratory disease disparities) (Wang *et al* 2023). Successful environmental interventions in Jordan require not only technical solutions but also institutional coordination and monitoring (Al-Shurafat *et al* 2021).

Jordan’s BC emission challenge reflects systemic constraints shared by arid regions globally: fossil fuel dependence, limited mitigation capacity, and competing water-energy-climate pressures. Elevating BC from a secondary concern to a central component of integrated air quality-climate policy would enable Jordan to demonstrate co-benefit optimization applicable to similar contexts. Effective mitigation requires replacing incremental measures with coordinated, multisectoral interventions targeting energy, transport, and land-use systems simultaneously —where desertification control, electric mobility, and regional climate diplomacy converge. As global attention pivots to methane and CO_2_, Jordan has an opportunity to lead on BC, transforming a hidden threat into a catalyst for innovation.

## 5. Limitations and uncertainty

In this section, we provide narrative and quantitative explaining how key modeling assumptions—such as population, GDP growth rates, BC GWPs, activity levels, fuel consumption, and emission factors—introduce uncertainties on BC estimation, highlighting how variations in key parameters could influence overall estimates. We acknowledge inherent uncertainties associated with these assumptions; for example, population growth may deviate from baseline projections due to migration trends or economic fluctuations, and EFs can vary depending on fuel quality and technology type. Socioeconomic projections—carry significant sensitivity to contextual volatility; refugee influxes may alter population trajectories by ±0.4%/yr (propagating to ±12% 2050 emissions), while fossil fuel price shocks could perturb industrial energy demand by ±7%.

Activity data followed Jordan’s official 2022 energy/non-energy statistics as received directly from national stakeholders, with future trajectories based on national GDP/population projections to 2050 by Jordan Department of Statistics. BC emissions from biomass combustion—such as residential heating and cooking, open waste burning, and agricultural residue burning—were not included in the current inventory due to limited data availability and the relatively low national reliance on biomass in Jordan. According to the Jordanian Department of Statistics in 2023, 99.5% of urban households and 98.8% of rural households primarily rely on clean fuels and technologies for cooking (DoS 2024). As well, according to the Jordan Energy Balance (MEMR 2023), biomass contributes to less than 1% of total national energy consumption, largely due to the country’s limited forest resources and its widespread use of liquefied petroleum gas (LPG), and electricity (MEMR 2023). As well, according to the Fourth National Communication Report , incineration and open burning of waste account for less than 1% of greenhouse gas emissions from the waste sector. Additionally, strict regulatory measures have been implemented to discourage biomass burning by individuals (MoEnv 2022a). Nonetheless, sporadic open burning activities, particularly of municipal waste containers at individual level and agricultural residues at farm level, do occur sporadically. While the exclusion of biomass combustion is unlikely to significantly impact on the national BC estimates, this remains a limitation. Future inventory efforts should consider incorporating proxy approaches to better capture potential contributions from biomass burning. On another activity level, cement industry activity data were excluded based on verified evidence from the Ministry of Industry that the sector’s has transitioned to clinker imports, so no significant fuel burning is ongoing.

EFs were held constant within upcoming years, which may not capture technological advancements over time, but their uncertainty, particularly for small-scale combustion—was mitigated via consensus-based vetting by 40+ national experts, replacing generic global defaults. Key uncertainties in BC EFs, as illustrated by the ranges presented in table 1, stem from multiple sources. The IPCC (2006) Tier 1 default values often lack regional specificity and may reflect outdated technologies (Eggleston *et al* 2006). Similarly, the EMEP/EEA (2016) Tier 1 EFs tend to generalize across technologies and are primarily derived from European contexts (EEA 2016), potentially limiting their applicability in other regions. The EFs reported by Bond *et al* (2004) are constrained by temporal relevance—being based on mid-1990s conditions in India—as well as geographic specificity. Additionally, these values often incorporate an intentional upward bias through the selection of upper-range estimates. Bond *et al* (2013) further highlight the inherent uncertainty in BC emission factors for contained combustion, estimating a possible range from –30% to +120%.

In our analysis, the LEAP-IBC model does not differentiate the toxicity of individual PM_2.5_ constituents such as BC, organic carbon (OC), dust, or sea salt. Instead, it employs a mass-based, population-weighted ambient PM_2.5_ exposure approach, applying integrated exposure–response (IER) functions (Burnett *et al* 2014, Cohen *et al* 2017) to estimate premature mortality. These IER functions are calibrated against total PM_2.5_ exposure from various sources, rather than source- or species-specific toxicity. Accordingly, when estimating the share of premature deaths attributable to BC, we assumed that BC contributes to health effects in proportion to its mass fraction of total PM_2.5_. While this simplification aligns with the LEAP-IBC framework, we acknowledge that toxicological literature suggests BC may have higher toxicity per unit mass compared to other constituents (Janssen *et al* 2011). Future work could benefit from integrating emerging evidence on differential toxicity across PM_2.5_ species, should the LEAP-IBC platform support such refinement.

The prioritization of mitigation measures was informed by structured stakeholder engagements, including Focus Group Discussions (FGDs) and workshops conducted over a 20-month period. While this process ensured institutional buy-in, we acknowledge that outcomes may reflect organizational priorities rather than technically optimal pathways. Furthermore, EFs and fuel consumption data were sourced from national authorities and validated by stakeholders, introducing inherent uncertainties from:(1) Methodological limitations in official data collection systems, (2) Potential overreporting biases given established consensus on air pollution severity, and (3) Contextual factors like visible soot deposition (building discoloration in Amman) and epidemiological evidence of respiratory morbidity. These uncertainties necessitate cautious interpretation of abatement projections.

BC emissions in CO_2_-equivalent units are calculated multiplying Physical BC Mass with GWP. Higher GWP values amplify BC emissions for the same physical mass. Uncertainty in GWP propagates proportionally to BC estimates. The primary drivers of uncertainty in BC emission estimates stem from two key factors: (1) Scope of Climate Effects, where *ARI-only* GWPs (Collins *et al* 2013, MoEnv 2022b), accounting solely for direct aerosol-radiation interactions, yield conservative BC estimates, while *Total effects* GWPs (Bond *et al* 2013), incorporating aerosol-cloud interactions (ACI) and albedo, increase BC emissions by 2.6–3.5×; (2) Temporal Focus, as 20-year GWPs (3–5× higher than 100-year values) amplify average BC emissions by approximately 3.5×.

The absolute uncertainty in BC emissions from the central values used for GWPs, expressed as CO_2_-equivalent, varies significantly with the chosen time horizon. The 20-year emission range (14,378 Gg) is 3.6 times wider than the 100-year range (3,990 Gg), reflecting greater short-term climate sensitivity and associated GWP variability. In contrast, relative uncertainty remains consistent across timeframes, with both horizons exhibiting similar coefficients of variation (CV ≈ 44.5%), see table 6 . This indicates proportional uncertainty is independent of the assessment period. Dominating this variability, total-effects GWP (incorporating aerosol-cloud interactions and albedo effects) drives 68% of the overall emission uncertainty. Notably, BC estimates derived from GWP substantially exceed those using ARI-only (aerosol-radiation interactions only) benchmarks: by 161% (6470 versus ∼2,480 GgCO_2_-eq) under a 100-year horizon and 167% (23,004 versus ∼8,626 GgCO_2_-eq) under a 20-year horizon.

Align GWP time horizons with policy objectives: 20-year GWPs are appropriate for near-term strategies (e.g., 2030 decarbonization targets), while 100-year GWPs suit long-term climate stabilization frameworks. Explicitly label GWPs as ‘*ARI-only*’ or ‘*Total Effects*’ to clarify the subset of climate impacts (e.g., aerosol-radiation versus aerosol-cloud interactions) represented in emission estimates. Adopting *ARI-only* GWPs (e.g., Ministry references) systematically underrepresents BC’s climate impact by 61%–68% compared to *Total Effects* GWPs. While *Total Effects* GWPs better reflect the current scientific consensus on BC’s net forcing, their inherent uncertainty necessitates explicit range reporting in policy applications.

**Table 6.** Key statistics and uncertainty drivers for black carbon emissions (GgCO_2_-eq) under 100-year and 20-year GWPs for the baseline year 2022.

Horizon	GWPs used	BC Range (GgCO_2_-eq)	Mean BC (GgCO_2_-eq)	SD (GgCO_2_- eq)	CV
100-year	Collins *et al* (2013): 345	2,480–6470	4,475	±1,910.23	44.1%
	Bond *et al* (2013): 900				
	Bond and Sun (2005): 680				
	Fuglestvedt *et al* (2010):460				
20-year	Collins *et al* (2013): 1,200	8,626–23,004	15,815	±6,700.30	44.9%
	Bond *et al* (2013): 3,200				
	Bond and Sun (2005): 2,200				
	Fuglestvedt *et al* (2010): 1,600				

## 6. Conclusion

This study presents the first comprehensive national black carbon (BC) inventory for Jordan, establishing a critical baseline for evidence-based policy development and revealing the urgent need for targeted mitigation strategies to address both climate and public health imperatives. Our analysis demonstrates that Jordan emitted 6,977 tonnes of BC in 2022 (95% uncertainty range: 2,582–7,460 tonnes), with emissions dominated by the transport sector (59% ± 8%) and energy sector (41% ± 8%), primarily driven by diesel combustion by 67% of national BC emissions. The large variability in BC’s CO_2_-equivalent estimates—ranging from 2,480 to 23,004 GgCO_2_-eq in 2022 and 6,034 to 55,970 GgCO_2_-eq by 2050—highlights the dominant role of GWP selection for BC, with GWP ranges from 1,200–3,200 (20 yr) and 345–900 (100 yr), in driving uncertainty in climate impact assessments.

The application of the LEAP-IBC framework with locally validated inputs represents a methodological advancement over global inventories, which systematically underestimate BC emissions for Jordan by 35%–85% by using too low emissions factors. This discrepancy, consistent across multiple countries, underscores the critical importance of bottom-up inventories that incorporate local fuel quality, combustion conditions, and technology distributions rather than relying on generic global defaults. While bottom-up approaches such as LEAP-IBC remain foundational, alternative frameworks—including top-down satellite-driven methods, field observations, hybrid modeling, and receptor-based analyses—offer complementary insights into BC emissions. Future research in Jordan could benefit from integrating these approaches to refine national inventories, reduce uncertainty, and strengthen the evidence base for air quality and climate policy.

Full implementation of identified mitigation measures could reduce BC emissions by up to 53% by 2050, yet current strategies treat BC as a side benefit. The transport sector offers the fastest near-term reductions through Euro VI vehicle standards, fleet upgrades and electrified transit, while long-term energy strategies require accelerated renewables and careful policy alignment to avoid BC-intensive pathways. Jordan should adopt a BC-specific mitigation framework, elevating BC to a primary policy target via integration into its NDCs to leverage climate finance. Over the long term, combining transport decarbonization with land-use and desertification control can deliver sustained climate, health, and environmental gains.

Several limitations warrant attention. The exclusion of biomass combustion, while justified by Jordan’s high clean fuel adoption rates (e.g. 99.4% for cooking), may still underestimate emissions from informal waste burning, wildfires, and crop residue combustion. Technology-specific emission factors were not disaggregated; future inventories should better capture fleet heterogeneity by vehicle class, engine age, and emission standard. The health assessment relied on mass-based PM_2.5_ exposure, which may underestimate BC’s higher toxicity, suggesting our mortality estimates are conservative. Finally, variation in Global Warming Potential (GWP) assumptions introduces large uncertainties, underscoring the need for standardized protocols and explicit reporting in national inventories.

## Data Availability

The data cannot be made publicly available upon publication because they are not available in a format that is sufficiently accessible or reusable by other researchers. The data that support the findings of this study are available upon reasonable request from the authors.

## References

[ercae1660bib1] Ababneh M, Alzubi M, Smadi T, Al, Author C, Akialddin Al Smadi T, Ieee M (2023). Engineering management for assessment of solar energy development (case study of Jordan). Journal of Advanced Sciences and Engineering Technologies.

[ercae1660bib2] Abu-Rumman G, Khdair A I, Khdair S I (2020). Current status and future investment potential in renewable energy in Jordan: an overview. Heliyon.

[ercae1660bib3] Alasmar R, Schwartz Y, Burman E (2024). Developing a housing stock model for evaluating energy performance: the case of Jordan. Energy Build..

[ercae1660bib4] Alrwashdeh S S, Alrwashdeh S S, Alsaraireh F M, Saraireh M A (2018). Solar radiation map of Jordan governorates. International Journal of Engineering and Technology.

[ercae1660bib5] Al-Shurafat A W, Talozi S, Alhrahsheh T, Zaid M, Paterson E, Benramdane A, Afaneh A (2021). Realizing integrated wastewater/greywater management in Jordanian public schools. Waterlines.

[ercae1660bib6] Baumgartner J, Zhang Y, Schauer J J, Huang W, Wang Y, Ezzati M (2014). Highway proximity and black carbon from cookstoves as a risk factor for higher blood pressure in rural China. PNAS.

[ercae1660bib7] Blanco-Donado E P, Schneider I L, Artaxo P, Lozano-Osorio J, Portz L, Oliveira M L S (2022). Source identification and global implications of black carbon. Geoscience Frontiers.

[ercae1660bib8] Bond T C (2013). Bounding the role of black carbon in the climate system: a scientific assessment. Journal of Geophysical Research: Atmospheres.

[ercae1660bib9] Bond T C, Streets D G, Yarber K F, Nelson S M, Woo J H, Klimont Z (2004). A technology-based global inventory of black and organic carbon emissions from combustion. Journal of Geophysical Research: Atmospheres.

[ercae1660bib10] Bond T C, Sun K (2005). ‘Can reducing black carbon emissions counteract global warming?. Environ. Sci. Technol..

[ercae1660bib11] Burnett R T (2014). An integrated risk function for estimating the global burden of disease attributable to ambient fine particulate matter exposure. Environ. Health Perspect..

[ercae1660bib12] CCAC (2023). Jordan - Deliver Policy Analysis and Recommendations on SLCP Mitigation [JO-22-001].

[ercae1660bib13] CCAC (2025). Black Carbon.

[ercae1660bib14] Cohen A J (2017). Estimates and 25-year trends of the global burden of disease attributable to ambient air pollution: an analysis of data from the global burden of diseases study 2015. Lancet.

[ercae1660bib15] Collins W J, Fry M M, Yu H, Fuglestvedt J S, Shindell D T, West J J (2013). Global and regional temperature-change potentials for near-term climate forcers. Atmos. Chem. Phys..

[ercae1660bib16] Crippa M, Janssens-Maenhout G, Dentener F, Guizzardi D, Sindelarova K, Muntean M, Dingenen R, Van and Granier C (2016). Forty years of improvements in European air quality: regional policy-industry interactions with global impacts. Atmos. Chem. Phys..

[ercae1660bib17] Dar-Mousa R N, Makhamreh Z (2019). Analysis of the pattern of energy consumptions and its impact on urban environmental sustainability in Jordan: Amman city as a case study. Energy, Sustainability and Society.

[ercae1660bib19] DoS (2016). Population Projections for the Kingdom’s Residents During the Period 2015–2050.

[ercae1660bib20] DoS (2024). Jordan Population and Family Health Survey 2023.

[ercae1660bib21] EDGAR (2022). Global Air Pollutant Emissions. The Emissions Database for Global Atmospheric Research.

[ercae1660bib22] EEA (2016). EMEP/EEA Air Pollutant Emission Inventory Guidebook -2016.

[ercae1660bib23] Eggleston H S, Buendia L, Miwa K, Ngara T, Tanabe K (2006). 2006 IPCC Guidelines for National Greenhouse Gas Inventories.

[ercae1660bib24] EPA US (2025a). https://epa.gov/air-emissions-inventories/air-pollutant-emissions-trends-data.

[ercae1660bib25] EPA US (2025b). https://epa.gov/ghgemissions/understanding-global-warming-potentials.

[ercae1660bib26] Evans M, Kholod N, Kuklinski T, Denysenko A, Smith S J, Staniszewski A, Hao W M, Liu L, Bond T C (2017). Black carbon emissions in Russia: a critical review. Atmos. Environ..

[ercae1660bib27] FAO and UNEP (2020). The State of the World’s Forests 2020: Forests, Biodiversity and People.

[ercae1660bib28] Fuglestvedt J S, Shine K P, Berntsen T, Cook J, Lee D S, Stenke A, Skeie R B, Velders G J M, Waitz I A (2010). Transport impacts on atmosphere and climate: Metrics. Atmos. Environ..

[ercae1660bib29] Ghana Environmental Protection Agency (2018). Ghana’s National Action Plan to Mitigate Short-Lived Climate Pollutants (SLCPs).

[ercae1660bib30] Hamasha K M (2021). The increasing trend of black carbon and organic carbon in Jordan during the Period of 2007 to 2018. Nat. Environ. Pollut. Technol..

[ercae1660bib31] Hamasha K M, Almomani M S, Abu-Allaban M, Arnott W P (2010). Study of black carbon levels in city centers and industrial centers in Jordan. Journal of Physics.

[ercae1660bib32] Hamasha K M, Arnott W P (2010). ‘Photoacoustic measurements of black carbon light absorption coefficients in Irbid city, Jordan. Environ. Monit. Assess..

[ercae1660bib33] Henze D K, Hakami A, Seinfeld J H (2007). Development of the adjoint of GEOS-Chem. Atmos. Chem. Phys..

[ercae1660bib34] Hoesly R, Smith S (2024). CEDS v_2024_04_01 release emission data. Zenodo.

[ercae1660bib35] Hussein T, Boor B E, dos Santos V N, Kangasluoma J, Petäjä T, Lihavainen H (2017). Mobile aerosol measurement in the eastern mediterranean - a utilization of portable instruments. Aerosol Air Qual. Res..

[ercae1660bib36] Hussein T, Juwhari H, Al Kuisi M, Alkattan H, Lahlouh B, Al-Hunaiti A (2018). Accumulation and coarse mode aerosol concentrations and carbonaceous contents in the urban background atmosphere in Amman, Jordan. Arabian J. Geosci..

[ercae1660bib37] Hussein T, Saleh S S A, dos Santos V N, Abdullah H, Boor B E (2019). Black carbon and particulate matter concentrations in eastern mediterranean urban conditions: an assessment based on integrated stationary and mobile observations. Atmosphere.

[ercae1660bib38] Hussey S J K, Purves J, Allcock N, Fernandes V E, Monks P S, Ketley J M, Andrew P W, Morrissey J A (2017). Air pollution alters staphylococcus aureus and streptococcus pneumoniae biofilms, antibiotic tolerance and colonisation. Environ. Microbiol..

[ercae1660bib39] Institute for Health Metrics and Evaluation (IHME) (2021). GBD Interactive Data Visuals.

[ercae1660bib40] IPCC (2014). The Fifth Assessment Report.

[ercae1660bib41] IPCC (2021). The Physical Science Basis, Contribution of Working Group I to the Sixth Assessment Report of the Intergovernmental Panel on Climate Change.

[ercae1660bib42] IRENA (2021). Renewable Energy Statistics 2021.

[ercae1660bib43] Janssen N A H (2011). Black carbon as an additional indicator of the adverse health effects of airborne particles compared with pm10 and pm2.5. Environ. Health Perspect..

[ercae1660bib44] Jin Y (2024). Measurement report: assessing the impacts of emission uncertainty on aerosol optical properties and radiative forcing from biomass burning in peninsular Southeast Asia. Atmos. Chem. Phys..

[ercae1660bib45] Jones M W, Peters G P, Gasser T, Andrew R M, Schwingshackl C, Gütschow J, Houghton R A, Friedlingstein P, Pongratz J, Le Quéré C (2023). National contributions to climate change due to historical emissions of carbon dioxide, methane, and nitrous oxide since 1850. Scientific Data.

[ercae1660bib46] Jordan Petroleum Refinery (2025). https://jopetrol.com.jo/EN/List/Fuel.

[ercae1660bib47] Klimont Z, Kupiainen K, Heyes C, Purohit P, Cofala J, Rafaj P, Borken-Kleefeld J, Schöpp W (2017). Global anthropogenic emissions of particulate matter including black carbon. Atmos. Chem. Phys..

[ercae1660bib49] Kuylenstierna J C I (2020). Development of the low emissions analysis platform—integrated benefits calculator (LEAP-IBC) tool to assess air quality and climate co-benefits: application for Bangladesh. Environ. Int..

[ercae1660bib48] Kuylenstierna J C I, Heaps C, Hicks K, Vallack H, Malley C (2017). The Long-range Energy Alternatives Planning—Integrated Benefits Calculator (LEAP-IBC).

[ercae1660bib50] Lam N L (2012). Household light makes global heat: high black carbon emissions from kerosene wick lamps. Environ. Sci. Technol..

[ercae1660bib51] Li Y, Henze D K, Jack D, Henderson B H, Kinney P L (2016). Assessing public health burden associated with exposure to ambient black carbon in the United States. Sci. Total Environ..

[ercae1660bib54] Liu J (2013). Framing sustainability in a telecoupled world. Ecology and Society.

[ercae1660bib52] Liu J, Cohen J B, Feng Y, Wang S, Qin K (2025). OMI-derived mass- and number-conserved estimation of black carbon emissions. Environmental Science & Technology Letters.

[ercae1660bib53] Liu J, Hong J, Mao F, Gong W, Shen L, Liang S, Chen J (2020). Impact of assimilating multi-source observations on meteorological and PM2.5 forecast over Central China. Atmos. Res..

[ercae1660bib55] Macro Trends (2025). Jordan Urban Population 1960–2025.

[ercae1660bib56] Mallak A M, Hamasha K M, Abdallah M J (2024). Black carbon concentration in the district of Ar-Ramtha, Northern Jordan, and its sources at seven wavelengths. Jordan Journal of Earth and Environmental Sciences.

[ercae1660bib57] Malley C S, Lefèvre E N, Kuylenstierna J C I, Haeussling S, Howard I C, Borgford-Parnell N (2023). Integration of short-lived climate pollutant and air pollutant mitigation in nationally determined contributions. Climate Policy.

[ercae1660bib58] MEMR (2023). Energy Balance Data 2022.

[ercae1660bib59] Ministry of Environment of Dominican Republic (2021). Assessment of Short-Lived Climate Pollutant Mitigation in the Dominican Republic: Recommendations for NDC Enhancement.

[ercae1660bib60] MoEnv (2017). Jordan’s First Biennial Update Report.

[ercae1660bib61] MoEnv (2020a). Jordan’s Second Biennial Update Report (SBUR).

[ercae1660bib62] MoEnv (2020b). Transport Sector Green Growth National Action Plan 2021–2025.

[ercae1660bib63] MoEnv (2022a). Jordan’s Fourth National Communication on Climate Change | United Nations Development Programme.

[ercae1660bib64] MoEnv (2022b). National Climate Change Policy of the Hashemite Kingdom of Jordan 2022–2050.

[ercae1660bib65] Morad M (2022). Jordan Public Transport Diagnostic and Recommendations.

[ercae1660bib66] Nakarmi A M, Sharma B, Rajbhandari U S, Prajapati A, Malley C S, Kuylenstierna J C I, Vallack H W, Henze D K, Panday A (2020). Mitigating the impacts of air pollutants in Nepal and climate co-benefits: a scenario-based approach. Air Quality, Atmosphere & Health.

[ercae1660bib67] Nazer H (2019). Development an Energy Benchmark for Residential Apartments in Amman.

[ercae1660bib68] Novakov T, Rosen H (2013). The black carbon story: early history and new perspectives. Ambio.

[ercae1660bib69] Ohata S (2021). Arctic black carbon during PAMARCMiP 2018 and previous aircraft experiments in spring. Atmos. Chem. Phys..

[ercae1660bib70] Paliwal U, Sharma M, Burkhart J F (2016). Monthly and spatially resolved black carbon emission inventory of India: uncertainty analysis. Atmos. Chem. Phys..

[ercae1660bib71] PTV group (2025). Transport Planning Software.

[ercae1660bib72] Rantanen M, Karpechko A Y, Lipponen A, Nordling K, Hyvärinen O, Ruosteenoja K, Vihma T, Laaksonen A (2022). The Arctic has warmed nearly four times faster than the globe since 1979. Communications Earth & Environment.

[ercae1660bib73] Saadeh R, Khader Y, Malkawi M, Allouh M Z (2022). Communicating the risks of air pollution to the public: a perspective from Jordan and Lebanon. Environmental Health Insights.

[ercae1660bib74] Saeeden M (2011). Sustainable Energy Mix and Policy Framework for Jordan.

[ercae1660bib75] Sandri S, Hussein H, Alshyab N (2020). Sustainability of the energy sector in Jordan: challenges and opportunities. Sustainability.

[ercae1660bib76] SEI, Climate Analytics, E3G, IISD and UNEP (2023). Phasing Down or Phasing Up? Top Fossil Fuel Producers Plan Even More Extraction Despite Climate Promises Production Gap Report 202310.51414/SEI2023.050.

[ercae1660bib77] SEI US (2025). LEAP: Low Emissions Analysis Platform.

[ercae1660bib78] Sheldon D, Skinner I, Taeger N, Mugume S (2022). Assessment of Climate Change Mitigation Potentials and Actions in Uganda’s Transport Sector - Changing Transport.

[ercae1660bib79] Shindell D T (2012). Evaluation of the absolute regional temperature potential. Atmos. Chem. Phys..

[ercae1660bib80] Stohl A (2015). Evaluating the climate and air quality impacts of short-lived pollutants. Atmos. Chem. Phys..

[ercae1660bib81] Sun T, Liu L, Flanner M G, Kirchstetter T W, Jiao C, Preble C V, Chang W L, Bond T C (2019). Constraining a historical black carbon emission inventory of the United States for 1960–2000. Journal of Geophysical Research: Atmospheres.

[ercae1660bib82] The World Bank (2020). https://climateknowledgeportal.worldbank.org/country/jordan.

[ercae1660bib83] UNCCD (2022). Global Land Outlook 2022: Land Restoration for Recovery and Resilience.

[ercae1660bib84] UNDP (2023). Jordan Moving Towards Environmental Sustainability.

[ercae1660bib85] UNEP and WMO (2011). Integrated Assessment of Black Carbon and Tropospheric Ozone.

[ercae1660bib18] van Donkelaar A, Martin R V, Brauer M, Hsu N C, Kahn R A, Levy R C, Lyapustin A, Sayer A M, Winker D M (2016). Global estimates of fine particulate matter using a combined geophysical-statistical method with information from satellites, models, and monitors. Environ. Sci. Technol..

[ercae1660bib86] Wang R (2012). Black carbon emissions in China from 1949 to 2050. Environ. Sci. Technol..

[ercae1660bib87] Wang W, Khanna N, Lin J, Liu X (2023). Black carbon emissions and reduction potential in China: 2015–2050. J. Environ. Manage..

[ercae1660bib88] WHO (2021). Global Air Quality Guidelines: Particulate Matter (PM2.5 and PM10), Ozone, Nitrogen Dioxide, Sulfur Dioxide and Carbon Monoxide.

[ercae1660bib89] WHO (2023). Household Air Pollution.

[ercae1660bib90] World Economic Forum (2024). Black Carbon Reduction: A Rapid Action Plan.

[ercae1660bib91] World Nuclear Association (2024). https://world-nuclear.org/information-library/country-profiles/countries-g-n/jordan.

